# Supplementary material for: Long-term healthcare provider availability following large-scale hurricanes: A difference-in-differences study
Source: PLoS One. 2020 Nov 24;15(11):e0242823. doi: 10.1371/journal.pone.0242823 (PMC7685502; doi:10.1371/journal.pone.0242823)
Supplement: S1 Table — (DOCX) [file pone.0242823.s001.docx]

S1 Table. Case and Control Counties

1. Hurricane Katrina

| Affected Parishes (n=16) | Control Counties (n=36) | |
| --- | --- | --- |
| All in Louisiana  St. Helena  Washington  Livingston  Tangipahoa  St. Tammany  Ascension  Assumption  St. James  St. John the Baptist  St. Bernard  Plaquemines  Jefferson  Lafourche  Terrebonne  St. Charles  Orleans | Richmond (GA)  Bibb (GA)  Clarke (GA)  Floyd (GA)  Macon (GA)  Dougherty (GA)  Richland (SC)  Spartanburg (SC)  Greenville (SC)  York (SC)  Anderson (SC)  Charleston (SC)  Horry (SC)  Sumter (SC)  Beaufort (SC)  Portsmouth (VA)  Sumner (TN)  Knoxville (TN) | Anderson (TN)  Roane (TN)  Washington (TN)  Williamson (TN)  Montgomery (TN)  Haywood (TN)  Madison (TN)  Craven (NC)  Gaston (NC)  Guilford (NC)  Alamance (NC)  Mecklenburg (NC)  Wake (NC)  Wayne (NC)  Buncombe (NC)  Duplin (NC)  New Hanover (NC)  Union (NC) |

1. Hurricane Sandy

| Affected Counties (n=8) | Control Counties (n=21) | |
| --- | --- | --- |
| All in New Jersey  Atlantic  Cape May  Essex  Hudson  Middlesex  Monmouth  Ocean  Union | Delaware (PA)  Lehigh (PA)  Lancaster (PA)  Berks (PA)  York (PA)  Bucks (PA)  New Castle (DE)  Seminole (FL)  Madison (AL)  Onondago (NY)  Monroe (NY) | Albany (NY)  Lorain (OH)  Summit (OH)  Chesterfield (VA)  Henrico (VA)  Bristol (MA)  Hampden (MA)  Essex (MA)  Plymouth (MA)  Anoka (MN) |
