# Supplementary material for: Long-term healthcare provider availability following large-scale hurricanes: A difference-in-differences study
Source: PLoS One. 2020 Nov 24;15(11):e0242823. doi: 10.1371/journal.pone.0242823 (PMC7685502; doi:10.1371/journal.pone.0242823)
Supplement: S2 Table — (DOCX) [file pone.0242823.s002.docx]

| S2 Table. Changes in Provider Ratios by County Among Counties Impacted by Hurricane Katrina And In-State Louisiana Controls | | | |  |
| --- | --- | --- | --- | --- |
|  | Study Time Period | |  |  |
| Provider Ratios | Pre-period (SE) | Post-period (SE) | Change (CI) | |
|  |  |  | No Covariates | With Control Covariates |
| Primary Care Physicians |  |  |  |  |
| *Difference-in-differences*  Short Term |  |  |  |  |
| Disaster Counties | 6.33 (3.17) | 5.80 (4.56) | 1.46 (-1.6, 4.5) | 1.8 (-0.82, 4.42) |
| Control Counties | 6.74 (3.52) | 4.76 (2.99) |  |  |
| *Difference-in-differences*  Long Term |  |  |  |  |
| Disaster Counties | 6.33 (3.17) | 5.72 (3.48) | 1.1 (-1.6, 3.7) | 1.4 (-0.98, 3.78) |
| Control Counties | 6.74 (3.52) | 5.05 (3.14) |  |  |
| *Difference-in-trends* |  |  |  |  |
| Disaster Counties | -0.232 | -0.116 | 0.04 (-1.3, 1.4) | 0.03 (-1.1, 1.2) |
| Control Counties | -0.0592 | 0.101 |  |  |
| Nurse Practitioners |  |  |  |  |
| *Difference-in-differences*  Short Term |  |  |  |  |
| Disaster Counties | 0.817 (0.66) | 1.48 (0.87) | -0.2 (-1.03, 0.63) | -0.08 (-0.92, 0.77) |
| Control Counties | 1.31 (1.25) | 2.17 (1.82) |  |  |
| *Difference-in-differences*  Long Term |  |  |  |  |
| Disaster Counties | 0.817 (0.66) | 2.39 (1.1) | -0.23 (-1.2, 0.73) | -0.12 (-1.1, 0.9) |
| Control Counties | 1.31 (1.25) | 3.11 (2.2) |  |  |
| *Difference-in-trends* |  |  |  |  |
| Disaster Counties | 0.08 | -0.294 | -0.05 (-0.45, 0.35) | -0.05 (-0.46, 0.35) |
| Control Counties | 0.05 | 0.311 |  |  |
| Medical Specialists |  |  |  |  |
| *Difference-in-differences*  Short Term |  |  |  |  |
| Disaster Counties | 12.3 (8.25) | 8.5 (9.84) | -0.21 (-7, 6.6) | 0.6 (-3.7, 4.8) |
| Control Counties | 8.3 (6.8) | 4.8 (6.2) |  |  |
| *Difference-in-differences*  Long Term |  |  |  |  |
| Disaster Counties | 12.3 (8.25) | 8.36 (9.0) | -0.47 (-7, 6.1) | 0.17 (-3.8, 4.1) |
| Control Counties | 8.3 (6.8) | 4.89 (6.3) |  |  |
| *Difference-in-trends* |  |  |  |  |
| Disaster Counties | 0.00 | 0.0423 | 0.03 (-3, 3) | -0.005 (-1.8, 1.8) |
| Control Counties | 0.11 | 0.035 |  |  |
| Surgeons |  |  |  |  |
| *Difference-in-differences*  Short Term |  |  |  |  |
| Disaster Counties | 3.97 (2.5) | 2.07 (2.2) | -0.36 (-2.2, 1.5) | -0.2 (-1.5, 1.1) |
| Control Counties | 2.91 (2.5) | 1.36 (1.9) |  |  |
| *Difference-in-differences*  Long Term |  |  |  |  |
| Disaster Counties | 3.97 (2.5) | 2.29 (2.41) | -0.2 (-2.1, 1.7) | -0.06 (-1.4, 1.3) |
| Control Counties | 2.91 (2.5) | 1.43 (1.89) |  |  |
| *Difference-in-trends* |  |  |  |  |
| Disaster Counties | -0.04 | 0.08 | 0.06 (-0.8, 0.9) | 0.05 (-0.5, 0.6) |
| Control Counties | -0.04 | 0.03 |  |  |
| Note: Provider rates are healthcare providers per 10,000 individuals in a county. Standard errors were heteroskedasticity robust. Control covariates included total population, race and ethnicity, median household income, sex, and total number of hospitals. Difference in differences time periods were 2004 vs 2007 for short term and 2004 vs 2010 for long term. Differences in trends time periods were 2001-2004 and 2007-2010.  VIF was under 10 for all difference-in-differences models.  *p<0.1 **p<0.05 | | | | |
