# Supplementary material for: Long-term healthcare provider availability following large-scale hurricanes: A difference-in-differences study
Source: PLoS One. 2020 Nov 24;15(11):e0242823. doi: 10.1371/journal.pone.0242823 (PMC7685502; doi:10.1371/journal.pone.0242823)
Supplement: Attachment — Submitted filename: PLOS Table of Revisions.docx [file pone.0242823.s003.docx]

| Table of Revisions | |
| --- | --- |
| Review | Reply to reviewers |
| Control variables should be clearly distinguished from treatment variables. Direct effects of treatment variables both with and without control variables must be reported. | We have now reported the results of the two models (one with no control covariates included and the second with all control covariates included) in Table 2 and Table 3. |
| How do treatment effects change with the addition of control variables, and why? | For both Hurricane Katrina and Sandy, the magnitudes are quite stable with and without covariates. Additionally, the estimated parameters are consistent, especially for Hurricane Katrina. We have updated Table 2 and Table 3 to show the estimated parameters with and without covariates. |
| Standard statistical tests on multi-collinearity, misspecification, auto-correlation etc. need to be reported. | For each model, we ran a test of heteroskedasticity and a test of variance inflation factor. VIF was under 10 for all DiD models. There was some correlation in the difference-in-trends models (i.e. median household income and % black, as well as, total population and # hospitals). Robust standard errors were used for all models. We did not do a test of autocorrelation as we only have two values (one pre and one post) in our DiD model. We checked for misspecification by comparing models with and without covariates. Again, the magnitudes are quite stable and the estimated parameters are consistent, even with the addition of control variables. |
| The discussion section needs to elaborate model limitations that might arise due to biases induced by model specification, multi-collinearity etc. | One limitation of our study is that our dataset could have some inaccurate address information, especially for providers that do not promptly update their current business address in the UPIN/NPI database. Additionally, if a provider did not have a UPIN or NPI then they would not be included in our analysis. More information about our model is provided in the Supplemental Technical Appendix. |
| Did any of the affected counties have missing/0 providers during the study period for any of the provider categories? | None of the affected or control counties had missing data for providers during the study period in our dataset. There were no counties that had 0 providers for Hurricane Sandy. However, some Louisiana parishes (i.e. counties) did have 0 providers during the study period. They were as follows:   - St. Charles Parish   - There were 0 Nurse Practitioners in 2001 and 2002 - Plaquemines Parish   - There were 0 Nurse Practitioners in 2001   - There were 0 Medical Specialists in 2007, 2009, 2010   - There were 0 Surgeons in 2007, 2009, 2010 - St. James Parish   - There were 0 Surgeons in 2009 and 2010 - St. Helena   - There were 0 Surgeons in 2004 and 2006 - St. Bernard Parish   - There were 0 Surgeons in 2007, 2009, and 2010   One explanation for certain parishes not having surgeons might be that surgeons work at multiple hospitals; however, in the UPIN/NPI databases they are only allowed to list one business address as their primary place of practice. Due to how we cleaned the data, we only used the zip codes from a provider’s primary business address. |
| How did you identify counties that had a “sizeable influx of Katrina migrants”? This was listed as an exclusion criteria for control counties. | We reviewed peer-reviewed literature, key government whitepapers, and gray literature to determine which counties had the largest influx of Katrina migrants. We excluded all counties in Louisiana, Mississippi, and Alabama because they were the three states most affected by Hurricane Katrina. Cities that experienced similar population declines due to either Hurricane Katrina or Rita were Gulfport-Biloxi and Pascagoula (MS), Lake Charles (LA), and Beaumont- Port Arthur (TX). Of the individuals that evacuated, about 71% returned to their homes by October 2006. About 8% of Louisiana’s population in 2005 had permanently left the State. Some of the papers that we consulted to determine these counties are listed below:   - Fussell, E., Curtis, K.J. and DeWaard, J., 2014. Recovery migration to the City of New Orleans after Hurricane Katrina: a migration systems approach. *Population and environment*, *35*(3), pp.305-322. - [Groen, J.A. and Polivka, A.E., 2008. Hurricane Katrina evacuees: who they are, where they are, and how they are faring. *Monthly Lab. Rev.*, *131*, p.32.](https://www.bls.gov/opub/mlr/2008/03/art3full.pdf) - Groen, J.A. and Polivka, A.E., 2010. Going home after Hurricane Katrina: Determinants of return migration and changes in affected areas. *Demography*, *47*(4), pp.821-844. - United States. Executive Office of the President, Etats-Unis. Assistant to the President for homeland security, counterterrorism, Superintendent of Documents, President of the United States Staff, United States. Assistant to the President for Homeland Security, Counterterrorism and Superintendent of Documents Staff, 2006. *The federal response to Hurricane Katrina: Lessons learned*. Government Printing Office.   We have provided more in-depth information on our model and county case selection in the Supplemental Technical Appendix. |
| The methods stats that control counties were randomly selected and determined not to be statistically significantly different in sociodemographics than affected counties, but in Table 1 there is a significant difference in county population between control and affected counties before the hurricane Katrina. | While the mean population is statistically significantly different between affected and unaffected counties for Hurricane Katrina, the population growth between the two groups is not statistically significant.  Due to our decision to limit control counties to the South, we had a limited number of counties that we could include that had similar socio-economic demographics. Additionally, many counties that were rural and coastal experienced other hurricanes during the study period and counties that were more urban often had a large influx of Katrina refugees. |
| Why is the term parish used sometimes instead of county? Is that a meaningful distinction? | The term parish is used by the State of Louisiana in place of the word county. Parishes and counties are considered equivalent. We have noted this in the manuscript. |
| The authors hypothesize that the decrease in providers after Katrina may be due to providers migrating. A potential driver could be hospital closures, which then force providers to look elsewhere for work. They have number of hospitals by county in Table 1 – does that number change following Katrina? | The 2017-2018 AHRF dataset only provided hospital data as far back as 2010. We agree with the reviewer that hospital closures could be a potential, even likely, driver that forces certain providers to look elsewhere for work. For example, Memorial Hospital in New Orleans has still not reopened to this day. Additionally, a 2006 report by the GOA notes that multiple hospitals were closed in Orleans, Jefferson, and St. Bernard Parishes. - <https://www.gao.gov/assets/260/251950.pdf> We used historic data from 2001-2017 from the American Hospital Association annual survey to calculate the total number of hospitals in the United States by county via FIPS codes. The hospital numbers in each FIPS code include community hospitals, federal government hospitals, nonfederal psychiatric hospitals, and other hospitals (i.e. nonfederal long term care hospitals and hospital units within an institution such as a prison or school infirmary). The mean number of hospitals in treatment and control counties are reported in the table below. Year \| Affected by Hurricane Katrina \| Unaffected by Hurricane Katrina \| Orleans Parish; 2004 \| 3.6 \| 3.6 \| 16; 2007 \| 3.8 \| 3.8 \| 10; 2010 \| 4 \| 3.7 \| 9; 2015 \| 3.8 \| 3.6 \| 10 |
| Is there a threshold that is used to determine whether an area is officially determined to have a provider shortage? If so, do the declines following Katrina meet this threshold? A decline on its own does not necessarily indicate a shortage. | We have addressed this in the discussion, referencing primary care physician supply and demand projections from:  National Center for Health Workforce Analysis Bureau of Health Workforce Health Resources and Services Administration U.S. Department of Health and Human Services. State-Level Projections of Supply and Demand for Primary Care Practitioners: 2013-2025 Washington, D.C.: U.S. Department of Health and Human Services, 2016. |
| Given the similar results for the short term and long term DID analyses, it seems the impact of disasters on healthcare provider availability is relatively permanent/stable. Perhaps more discussion on this is warranted. Was there any targeted intervention attempted after Katrina to recruit healthcare providers back to the area? | This is an excellent point, thank you. We have included references to the efforts that were made to address recruitment after Hurricane Katrina in the discussion section. |

Mean Number of Hospitals

|  | Disaster | Control | Orleans Parish |
| --- | --- | --- | --- |
| 2001 | 3.25 | 3.5 | 16 |
| 2002 | 3.25 | 3.5 | 16 |
| 2004 | 3.5625 | 3.55556 | 16 |
| 2007 | 3.8125 | 3.80556 | 10 |
| 2009 | 4.0625 | 3.75 | 10 |
| 2010 | 4.0625 | 3.69444 | 9 |
